# Long term patient perspectives following all types of bariatric surgery: A 19-year follow-up study

**DOI:** 10.1016/j.obpill.2026.100260

**Published:** 2026-03-20

**Authors:** G. Konings, M. Drukker, R. Severeijns, R. Ponds

**Affiliations:** a Department of Psychiatry and Neuropsychology, Mental Health and Neuroscience Research Institute (MHeNs), Maastricht University, the Netherlands; b Department of Medical Psychology, Maastricht University Medical Centre (MUMC), Maastricht, the Netherlands; c Department of Medical Psychology, Amsterdam University Medical Centre (UMC), Amsterdam, the Netherlands

**Keywords:** Bariatric surgery, Health behaviors, Long-term outcomes, Patient perspective

## Abstract

**Background:**

Bariatric surgery effectively treats severe obesity. However, long-term data on weight maintenance and patient well-being remain scarce. This study assessed patients' perspectives on their bariatric trajectory and outcomes of their historical and current status across multiple domains, irrespective of surgical procedure.

**Methods:**

A retrospective cohort study was conducted among patients who were referred to the Department of Medical Psychology for bariatric screening between 1998 and 2004. Of the 547 referred patients, eligible participants received a survey after informed consent addressing weight outcomes, current eating patterns, dietary guidelines adherence, expectation fulfillment, satisfaction, and peer advice.

**Results:**

Sixty-two participants completed survey. Mean postoperative time since the initial surgery was 19 years (range 6–27). Mean preoperative weight decreased 33%, from 144.2 to 97.5 kg. Preoperative Body Mass Index decreased from 50.3 to 34.0 kg/m^2^. Healthy postoperative behaviors included regular meals, portion control and control regarding snacking between meals. Unhealthy behaviors included evening snacking, alcohol consumption and binge eating. One third reported using vitamins never or sometimes. Participants reported diverse encountered barriers regarding eating, drinking, physical and psychological health and social functioning. Most would choose surgery again except those with expectation-outcome discrepancies or ongoing weight instability. Peer advice emphasized comprehensive preoperative education on all potential postoperative consequences from multiple sources.

**Conclusion:**

After 19 years, weight loss, healthy eating and adherence to advice varied, yet most participants were satisfied regardless of weight loss or reoperation. We plea for lifelong follow-up care to optimize bariatric results regardless type of surgery.

## Highlights

• After 19 years, most patients would choose bariatric surgery again, with high satisfaction irrespective absolute weight loss.
• Weight stability, not absolute weight loss, is what matters most for long-term patient satisfaction.
• Discrepancies between preoperative expectations and actual outcomes were key predictors of dissatisfaction.
• One-third of patients discontinue vitamin supplements long-term, risking preventable nutritional complications.

## Graphical abstract

**Figure:**
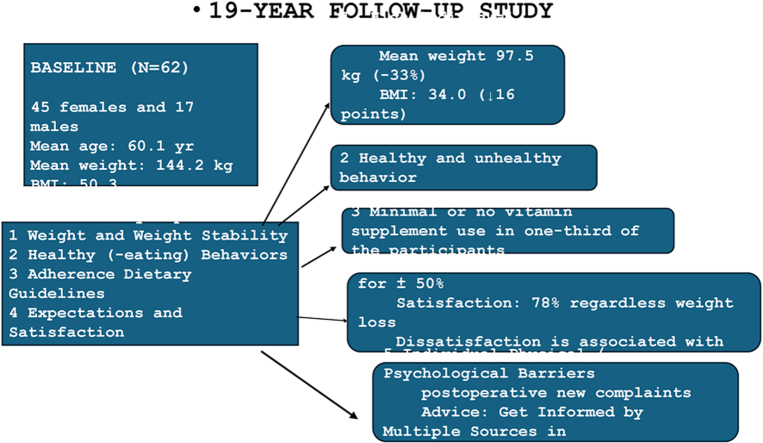

## 1 Introduction

Bariatric surgery produces substantial weight loss and improves comorbidities, particularly when compared to nonsurgical interventions that rarely maintain results beyond 2 years [[1], [2], [3]]. Since the early weight loss procedures, various bariatric techniques have been developed, utilizing malabsorption, restricted intake or both. Initial procedures such as the vertical banded gastroplasty (VBG) were succeeded by procedures including Laparoscopic Adjustable Gastric Banding (LAGB) and Laparoscopic Roux-en-Y Gastric Bypass (LRYGB). However, weight regain remains common, even with currently popular types as Laparoscopic Gastric Bypass (LGB) and Laparoscopic Sleeve Gastrectomy (LSG) [4].

Despite advances in the bariatric field, knowledge gaps exist, particularly regarding long-term outcomes. Extended follow-up studies are essential to comprehensively evaluate the impact of surgery throughout a patients' lifespan. While physiologic mechanisms induced by surgery facilitate changes in eating behaviors, these mechanisms attenuate over time and do not guarantee sustained lifestyle modifications [5]. Studies exceeding ten years post-surgery are limited, with most research covering maximum three-year follow-up periods [2] and fewer examining five-to ten-year outcomes. Long-term research is known to present methodological challenges including participant attrition, lack of control data and funding constraints [2].

The increasing prevalence of bariatric surgery has been accompanied by rising reoperation rates, primarily to address insufficient weight loss or complications. While durable weight loss represents a key outcome criterion [3], from a patient's perspective, bariatric surgery involves multiple domains beyond weight and comorbidities [6] with issues of adjustments to dietary guidelines, management of food and eating behaviors which are essential for weight loss maintenance [7]. Recommended eating behaviors after surgery include consuming small bites, distributing food intake across 4–6 daily meals, thorough chewing in a relaxed manner and terminating meals upon feeling “comfortably full” [8]. Recommendations include no alcohol consumption and the necessity of daily vitamin supplementation [8]. To comprehensively evaluate the efficacy of bariatric surgery, the patient's perspective should be incorporated [9]. Valuable outcomes identified by patients differed from those emphasized by bariatric providers [9]. While certain outcomes such as diabetes remission represented shared priorities, others as excess skin and quality of life measures demonstrate discordance [9]. Comprehensive information regarding the multifaceted postoperative impact is crucial for enhancing patient decision-making [4] and requires continuous updating including the relationship between preoperative expectations and postoperative outcomes [3].

In a previous publication, we presented the patients' perspective five years after bariatric surgery regarding weight loss, eating behaviors and dietary guidelines compliance [10]. The primary objective of the present study was to explore the patients’ bariatric trajectory and perspective beyond five years regardless type of surgery. The survey investigated weight loss, weight stability, surgical history regarding type and frequency of surgery, current eating behavior (meal regularity, snack control, evening/night eating) and adherence to advised dietary guidelines (meal distribution, fluid consumption with meals, alcohol consumption, vitamin supplements). Additional questions assessed expectation fulfillment, encountered challenges, hypothetical reconsideration of bariatric intervention and advice for prospective bariatric candidates. The focus was on patient experience with bariatric surgery broadly, not procedure-specific outcomes. The examined aspects were expected to be transferable across surgical techniques since all patients were related to fundamental challenges of living with a surgically altered digestive system.

## 2 Research methodology

This study employed a cross-sectional design examining patient-reported outcomes approximately 19 years after bariatric surgery and represents a separate cohort from our previously published 5-year follow-up study [11]. The Medical Ethics Committee (MEC) of Maastricht University Medical Centre (MUMC+) approved this study. All procedures complied with the current version of the Declaration of Helsinki.

### 2.1 Participants and procedure

In 2020, the MUMC + Department of Psychology filed records for 547 patients referred for bariatric screening between 1998 and 2004. Due to General Data Protection Regulation constraints, addresses could not be updated. It was estimated that a substantial portion of the address database was likely to be inaccurate, as there was a high probability that individuals had relocated during the approximately 19 years. With MEC approval, each address received an informative letter. The correspondence solicited participation in a research study, specifically targeting individuals with prior experience in weight-reduction surgery who were willing to share their personal experiences. Written informed consent was obtained from all individual participants included in the study. All types of bariatric surgery were included in this study. Two months after the first letter, a reminder was sent. No incentives were provided. Consenting participants received an electronic survey link or a paper survey with return postage.

### 2.2 Survey instrument

The survey collected data on type and frequency of surgery, pre- and postoperative weight, weight stability, height, eating behaviors, compensatory mechanisms and adherence to dietary guidelines. Height and weight data were used to calculate BMI by means of the formula: weight in kilograms/the square of height in meters. Weight loss was reported as percentage weight loss and as percentage of excess weight loss (%EWL), calculated using an ideal body weight equivalent to a BMI of 25 kg/m^2^, with %EWL defined as the loss of excess weight above this threshold expressed as a percentage of initial weight [2].

Weight regain nearly to preoperative levels was defined as ≤5% difference from baseline [3]. Weight stability was defined as weight fluctuations of approximately 3 kg or less, consistent with an established criteria of <2.5 kg variation per month over three consecutive months while weight instability was defined as fluctuations exceeding 3 kg [12]. In the survey, weight stability was patient-defined as weight fluctuations of approximately 3 kg or less, a threshold that distinguishes stable weight maintenance from meaningful fluctuations in a manner accessible to patients without detailed weight records. Participants reported weight stability duration in years and potential contributing factors to instability.

Eating behavior assessment included meal regularity, between-meal snacking control, evening (20:00–00:00) and nighttime (00:00–06:00) eating patterns, loss of control experiences and binge eating including frequency per week.

Dietary guideline adherence assessment included meal distribution throughout the day, meal skipping, fluids consumption during meals, alcohol consumption, and vitamin supplements. Participants indicated whether their current eating behaviors were responsible for stable weight, contributed to weight regain, or facilitated additional weight loss. Dissatisfied participants specified desired behavioral modifications via open-ended responses.

Compensatory behavior assessment included restrained eating, laxative/diuretic use, self-induced vomiting and excessive exercise.

Eating behavior and dietary guideline adherence items utilized a 4-point Likert scale ('never,' 'sometimes,' 'often,' or 'always'). Compensatory behaviors were assessed dichotomously (yes/no). Binge eating frequency was quantified as the number of episodes per week. Expectation fulfillment was assessed by means of an open-ended question: “Did preoperative expectations match the postoperative results?” including the opportunity to specify rationale.

Encountered challenges were assessed by means of an open-ended question: “What kind of encountered barriers were encountered?”. Advice for prospective bariatric patients was assessed by means of the question: “What advice would you have for patients who consider bariatric surgery?”. Satisfaction was assessed through willingness to hypothetically undergo bariatric surgery again given current knowledge, with opportunity to specify rationale.

### 2.3 Statistical analyses

The aim of this study was to examine the patient experience after bariatric surgery across a broad range of topics. Analyses were conducted using STATA, version 13, with statistical significance set at p < 00.05. Descriptive statistics characterized eating behaviors, dietary guideline adherence, expectations, satisfaction, and peer advice. Paired t-tests analyzed pre-post BMI differences. Independent t-tests examined associations between weight instability and weight loss, satisfaction and weight loss, and surgical frequency and weight loss. Chi-square analyses assessed associations between weight instability and sex, weight instability and satisfaction, and preoperative expectations and satisfaction.

## 3 Results

### 3.1 Demographic data

Table 1 summarizes all response and non response data. Over half did not respond (60%) and one third turned out to have moved and not traceable. Of all eligible responses (n = 102), 78 participants consented to participate. Sixty-two participants (45 females, 17 males) of the 102 eligible participants completed all questions (response rate: 61%). Mean age was 60.1 years (sd = 8.0; range 39–76). Mean time since initial surgery was 18.7 years (sd = 3.7; range 6–27).

**Table 1:** Overview of responses.

*Registry of referrals to the department of Psychology for pre-bariatric surgical assessment 1998-2004*	N (%)
Informational correspondence regarding the study and invitation for participation	547 (100)
Non-respondent cases	329 (60)
Responses (all types)	218 (40)
Responses indicating study ineligibility	
Subject relocation to alternative domicile	71 (33)
Subject did not undergo bariatric intervention	16 (7)
Mortality notification	29 (13)
Responses meeting inclusion criteria	
Eligible subjects declining participation	24 (11)
Subjects providing informed consent	78 (36)

Subjects completing assessment instruments (100% completion rate)	62
Subjects with incomplete assessment data (<50% completion rate)	16

### 3.2 Pre and postoperative weight

Table 2 presents weight and BMI outcomes. Mean weight decreased by 33% from 144.2 kg (sd:31.1) 97.5 kg (sd: 21.3). Mean BMI decreased from 50.3 (sd:10.0) to 34.0 (sd:6.7). Preoperative BMI ranged from 36.8 to 80.8 points while postoperative BMI ranged from 22.2 to 49.5 points. Mean EWL was 61.2% (sd:32.8).

**Table 2:** An overview of mean weight and BMI, frequency of surgery and weight (in-) stability.

	N	Mean weight (kg.) (sd; range)	Mean BMI (sd; range)
Before surgery	62	144.2 (31.1; 98–240)	50.3 (10.0; 36.8–80.8)
At time of the study	62	97.5 (21.3; 61–145)	34.0 (6.7; 22.2–49.5)
Subgroup with 1 surgery	27	102.9 (21.5; 66–145)	36.1 (6.8; 25.0–45.8)
Subgroup with 2 surgeries	27	93.5 (20.6; 62–140)	32.0 (5.7; 22.2–47.3)
Subgroup with 3 surgeries	7	92.4 (21.1; 61–130)	34.0 (8.7; 23.8–49.5)
Weight stability subgroup^a^	37	90.1 (17.3; 61–123)	31.4 (5.5; 22.2–45.7)
Weight instability subgroup^a^	25	108.2 (22.2; 68–145)	37.8 (6.6; 25.0–49.5)

a Postoperative weight of participants who reported weight stability versus postoperative weight of participants who reported weight instability.

Forty-four percent of the participants underwent one surgery. Over half of the participants underwent two (44%) or three surgeries (12%). Table 2 presents mean weight per frequency of surgeries. Mean postoperative weight for single-surgery participants was 102.9 kg (sd = 21.5; range 66–145 kg) versus 93.3 kg (sd = 20.4; range 61–140 kg) for multiple-surgery participants. Preoperative weights were similar between groups: 149.3 kg (sd = 36.7) and 140.2 kg (sd = 25.9), respectively (t = 1.13, df = 59, p = 0.26). Weight loss did not significantly differ based on surgical frequency (t = 1.78, df = 59, p = 0.08).

### 3.3 Weight stability

Sixty percent of the participants reported weight stabilization for a mean duration of 7.7 years (sd:5.4, range: 1–18 years) with no significant sex differences (chi-square = 0.11, p = 0.741, df = 1). The weight-stable subgroup demonstrated significantly greater weight loss than the weight-unstable subgroup (t = 3.59, df = 59, p < 0.001), despite similar preoperative weights: 147.9 kg (sd = 34.6) versus 141.6 kg (sd = 28.7; t = 0.77, df = 59, p = 0.44). Compared to weight-stable participants, weight-unstable participants reported significantly greater discrepancies between preoperative expectations and postoperative outcomes (chi-square = 5.00, p = 0.025, df = 1) and reduced satisfaction with their bariatric trajectory (chi-square = 5.197, p = 0.0253, df = 1). Reported weight instability contributors included snacking, emotional/restrictive eating, technical complications (displaced/removed gastric bands), health issues (knee problems, reflux, diaphragmatic hernia), mobility limitations, and significant life events (divorce).

### 3.4 Surgical procedures and reoperation rate

Initial procedures included predominantly LAGB (49%), VBG (37%) or LRYGB (9%). Secondary procedures predominantly involved LRYGB (63%). Tertiary interventions included LRYGB or a duodenal switch procedure.

### 3.5 Reported health behaviors and dietary guideline adherence

Table 3 presents current eating behavior, dietary guideline adherence and compensatory mechanisms categorized as healthy (H) or unhealthy (U). After surgery, most commonly reported healthy eating behaviors included: regular meals and portion control. Unhealthy behaviors such as loss of control eating, evening eating, and between-meal snacking persisted with varying frequency. Approximately one-third of participants (32%) reported minimal or no control regarding snacking between meals (never or sometimes), while the majority (68%) reported control often or always. Therefore, for some, snacking between meals remained a challenging area for long-term behavior modification.

**Table 3:** Frequency of (un-) healthy behavior and adherence to advised guidelines.

N = 62	Current behavior				H/U^b^
*Eating behavior (%)*	*never*	*Some-times*	*often*	*always*	
Regularity of meals	5	10	50	35	H
Portion control per meal	2	12	42	45	H
Control over snacking between meals	2	30	47	22	H
Loss of control food consumption^a^	45	43	10	2	U
Evening eating	33	50	12	5	U
Night-time eating	95	5	–	–	U

*Adherence to guidelines (%)*					
Distribution nutrient intake	17	18	33	32	H
Small portions per time	10	20	30	40	H
Fluids during meals	27	32	27	15	U
Alcohol consumption	57	38	5	0	U
Skipping meals	47	42	10	2	U
Vitamin supplementation	22	10	13	55	H

*Compensatory mechanisms (%)*	*Yes %*				
Restrained eating1a	4				U
Laxatives1a	2				U
Diuretics1a	0				U
Self-induced vomiting1a	4				U
Excessive exercises/sports1b	5				U
Objective binge eating episodes	17				U

1a Twice per week for at least 3 months.

1b At least three times per week for at least 3 months to reduce weight.

a At least two times per week loss of control regarding eating.

b Behaviors are classified as healthy (H), recommended for optimal outcomes or unhealthy (U) associated with increased risks.

Nighttime eating was uncommon. Binge eating decreased significantly but was not completely resolved in all participants. Preoperatively, 55% of the respondents reported objective binge eating episodes (mean = 4.8 weekly, sd = 2.8, range 1–10) compared to 17% at the time of the survey (mean = 1.4 weekly, sd = 0.7, range 1–3; t = 5.67, df = 59, p < 0.001). Most participants (68%) were satisfied with their current eating behaviors and believed these behaviors maintained their weight (73%). By means of an open-ended question, dissatisfied participants could specify what needed to be changed. Needs for improvement were: more meal regularity, healthier food choices and enhanced control over sweets, snacks and evening eating. Weight loss did not significantly differ between satisfied (47.9 kg, sd = 31.2) and dissatisfied participants (40.8 kg, sd = 33.3; t = −0.71, df = 58, p = 0.48).

High adherence to postoperative dietary guidelines was reported for meals distribution throughout the day (65%) and small meals per time (70%). Approximately half of the participants never skipped meals, consumed liquids during meals or drank alcohol. Infrequently reported unhealthy behaviors (<10%) included night time uncontrolled eating and compensatory behaviors.

### 3.6 Expectations and satisfaction

For 45% of the participants, preoperative expectations aligned with postoperative results. The remaining 55% reported outcomes exceeding or falling below expectations, citing increased energy, greater weight loss and enhanced physical capacity than anticipated or persistent fatigue, insufficient weight loss, food intolerances, excess skin and inadequate aftercare. Table 4 includes participant responses to the open-ended question regarding whether their preoperative expectations were met.

**Table 4:** Responses after open-ended questions expectation, encountered barriers and advices for peers.

Participants' reponses regarding:	
Expectations*	*Surgery*
	+ For me surgery was the start of a new life
	+ I would elect surgery again but would opt for an alternative procedure
	- Complications after surgery were more worse than my weight
	- Aftercare was inadequate and should be much more
	*Health (physical and mental)* + I am happy with my current weight of 90 kg.
	+ My self esteem has improved
	- I did expect more results, I still feel tired and without energy
	- I suffer from dumping syndrome and gastric reflux which has impact on my social life - I am back to square one
	*Weight loss* + My weight now is healthier
	+ without surgery my weight now would be worse
	- I did expect more weight loss - due to weight loss I suffer from excess skin
	*Complications* - I have problems with eating, vomiting and being nausea
	- My belly is full of scar tissue. Surgery is no option anymore

Encountered Barriers*	*Eating and drinking* - Food intolerance (for example rice, meat) - Eating and drinking results in vomiting and diarrhoea *Physical issues* - Excessive skin due to weight loss including blemishes - Tiredness, low energy, lack of a variety of vitamins
	*Psychological issues* - Loneliness due to lack of social understanding and support
	- Feeling ashamed due to weight regain *Social life* - Challenges in social life as eating outdoors and pubs
	- Comments and blaming by others regarding weight loss and weight regain

**Advices for peers**	Get informed by multiple sources (professionals, peers, books)
	Use social support to persevere
	Think twice, it will change your daily life forever
	Keep in mind: surgery works out different for everyone
	Surgery is an aid, not a simple solution
	You are the one who has to do the changes
	Be aware of all kinds of consequences
	Get coached before and after surgery
	Comply to the advised dietary guidelines to reduce risks
	Be honest with yourself and mind your expectancies
	Beware of emotional eating
	Do not choose a bariatric route to reduce weight
	Apply when there are no alternatives left

* + : positive comment -: negative comment.

Expectation-outcome discrepancies were associated with reduced weight loss approaching significance (t = −1.99, df = 58, p = 0.05) and significantly lower likelihood of hypothetical surgical reconsideration compared to participants reporting expectation-outcome congruence (t = 2.51, df = 58, p = 0.01).

Regarding satisfaction, 78% would hypothetically undergo bariatric surgery again, independent of weight loss. Mean weight loss amongst satisfied participants was 47.9 kg (sd:31.72) versus 40.8 kg (sd:33.3) amongst dissatisfied participants (t = −0.71, df = 58, p = 0.48). Satisfaction after one, two or three surgeries was respectively: 85%, 80% and 57%. No significant difference was found between single versus multiple surgery participants (t = 0.53, df = 58, p = 0.60) but weight-unstable participants were significantly less likely to reconsider bariatric surgery compared to weight-stable participants (chi-square = 5.19, p = 0.023, df = 1).

### 3.7 Encountered barriers and peer advice

Table 4 presents reported postoperative encountered barriers and advices for peers at the beginning of the bariatric journey. The encountered barriers were categorized into eating and drinking, physical, psychological and social domains, overlapping with expectations.

Two-third of all participants provided peer advices ranging from strong recommendations to cautionary guidance not to start a bariatric route. Most frequently advised was gathering comprehensive information from multiple sources regarding potential postoperative consequences. Additional recommendations highlighted awareness of emotional eating's impact, the importance of social support and necessity of dietary guideline adherence. Participants emphasized that bariatric surgery should be viewed as a facilitating tool for weight loss rather than a complete solution. They stressed that long-term success requires permanent lifestyle modifications. Many participants wanted prospective patients to understand that surgery is not a 'quick fix' but rather an intervention that requires sustained personal effort and commitment to achieve and maintain results.

Some participants reported loneliness due to lack of understanding from family and friends regarding their surgical decision and insufficient emotional support during the postoperative period, leaving them feeling isolated in their bariatric journey.

## 4 Discussion

The core objective of this study was to elicit patients' retrospective perspectives on their bariatric surgical trajectory and document their current and historical status across multiple long-term postoperative outcome domains, irrespective of surgical procedure.

### 4.1 Weight loss and weight stability

After 19 years, mean weight loss was 33% with BMI reduction of 16 points and EWL of 61.2%. Individual variability was substantial, ranging from 150 kg weight loss to 20 kg weight gain. These findings align with limited long-term studies beyond 10 years reporting 14% weight loss after LAGB, 25% after gastric bypass, and 16% after VBG, with EWL ranging from 27 to 69% across procedures [2,13]. A 20-year LAGB study reported mean weight loss of 22% (30.1 kg) and BMI reduction of 10.6 points [2]. Outcome variability has been attributed to surgical procedure, postoperative duration, and eating behaviors [14].

Weight stability was reported by more than half of the participants and was associated with greater weight loss and satisfaction. Conversely, weight-unstable participants demonstrated less weight loss and reduced satisfaction. To our knowledge, no previous study examined persistence of weight instability after surgery. The weight instability findings require replication but may reflect persistent preoperative patterns. Literature primarily focuses on postoperative weight regain, which remains a concern even with contemporary procedures [4]. Approximately 20% of patients experience over 15% body weight regain within 5 years, with 10-year data indicating mean regain of 36% (range 12–71%).

In this study, 6.5% of participants regained to preoperative weight, defined as ≤5% from baseline. Weight regain to nearly preoperative levels occurred in 3.3% of LRYGB, 12.5% of sleeve gastrectomy, and 36% of LAGB patients at five years [3]. Patients experienced weight regain as unexpected. They reported shame, discouragement, and frustration [15].

### 4.2 Surgical procedures and reoperation rate

Most participants underwent LAGB or VBG, procedures now obsolete or less common [16]. LAGB has declined despite fewer side effects, while VBG has been abandoned due to high failure and complication rates [2]. In the present data, contemporary procedures as LSG and LRYGB [3] were underrepresented. However, the examined aspects were expected to be transferable across surgical techniques since all patients face fundamental challenges of living with a surgically altered digestive system.

Over half of participants underwent second or third procedures. Reoperations are common across all procedures, with rates varying by type and postoperative duration [4]. LAGB 10-year reoperation rates ranged from 8 to 78% [3], while LRYGB rates ranged from 8 to 64% [[2], [3], [4]]. In this study, additional surgeries yielded no significant extra weight loss, with single-surgery participants demonstrating comparable outcomes. These results align with findings that reoperations five years post-surgery did not significantly impact weight loss or comorbidity remission [17]. Preoperative counseling regarding realistic reoperation expectations is warranted.

### 4.3 Health behaviors and dietary guideline adherence

Behaviors were classified as healthy (H), recommended for optimal outcomes or unhealthy (U) associated with increased risks. Percentages reflected actual patient-reported behaviors, not achievement of recommendations. Reported healthy behaviors were: regular meals, portion control and control regarding snacking between meals. Reported unhealthy behaviors were: meal skipping, uncontrolled eating, evening eating, alcohol consumption, and inadequate vitamin supplementation, mirroring our previous study, a cross-sectional cohort examining patient-reported outcomes approximately 5 years after bariatric surgery [11]. Adopting healthy postoperative behaviors remained challenging for a substantial proportion of participants. Both studies documented persistent unhealthy eating behaviors and vitamin supplementation non-adherence in approximately one-third of patients, demonstrating these represent chronic challenges requiring sustained intervention rather than temporary postoperative adaptation issues that resolve spontaneously.

Literature identifies uncontrolled eating as a primary factor in suboptimal weight loss and regain after surgery [18]. Post-surgery patients must modify eating patterns and adhere to dietary guidelines throughout their lifespan to optimize outcomes [19,20]. Consistent with other studies, unhealthy eating behaviors including uncontrolled eating and binge eating disorders persisted. Nearly one-fifth of postoperative patients with uncontrolled eating met food addiction criteria [21]. Research demonstrates reduced energy intake with high dietary adherence during the first two years [14,20], with eating primarily motivated by physical rather than emotional factors [2,19,20]. However, within five years, emotional and unrestricted eating increased [20], and after 10 years, reduced weight loss correlated with uncontrolled eating [5].

In the preoperative phase, formal DSM eating disorder diagnoses (Bulimia Nervosa, Binge Eating Disorder) were exclusion criteria for bariatric surgery. Therefore, no participants had diagnosed eating disorders at the time of surgery. However, subthreshold binge eating behaviors, not meeting full diagnostic criteria, were not exclusionary.

Nighttime eating (5%) and binge eating (17%) were relatively uncommon but may impede weight loss. Notably, binge eating prevalence decreased substantially from preoperative levels 55%–17% at 19 years. This reduction is particularly meaningful given that formal DSM eating disorder diagnoses (Bulimia Nervosa, Binge Eating Disorder) were exclusion criteria for bariatric surgery during the preoperative screening period. However, subthreshold binge eating behaviors not meeting full diagnostic criteria were not exclusionary, explaining the high preoperative prevalence.

Previous research reported nighttime eating prevalence of 6–8% up to eight years postoperatively, associated with increasing BMI, with 15–20% of nighttime eaters meeting binge eating disorder criteria [22]. These patient-centered findings highlight the importance of enhanced focus on long-term behavioral patterns.

### 4.4 Vitamin supplementation

Vitamin supplementation is a healthy recommended behavior to prevent adverse health consequences including nutritional deficiencies [4]. However, two-thirds of participants actually adhered to this recommendation, with one-third demonstrating unhealthy non-adherence, a concerning finding requiring clinical attention. Surgical techniques differentially affect nutrient absorption, potentially causing anemia, ataxia, hair loss, Wernicke encephalopathy [23] or increased fracture risk [24]. It has been shown that nutritional deficiencies occur in 30–70% of bariatric surgery patients as a result of reduced consumption or absorption [25]. During the first year after surgery, studies indicated moderate to high vitamin adherence [14,26] but over time, vitamin A and folate deficiencies increased [27]. After 17 years, an increased risk for anemia has been documented [28]. Postoperative monitoring remains critical [4,23,25,29], even after 19 years.

### 4.5 Alcohol

Over half of participants reported alcohol abstinence, while others reported occasional or frequent alcohol consumption. Postoperative alcohol avoidance is recommended due to potential B vitamin deficiencies and high caloric content [8]. Literature shows initial less alcohol consumption within the first two years after surgery but increased consumption thereafter, particularly among men [30]. Alcohol dependency risk has been observed compared to non-surgical controls [4], especially following RYGB whereas sleeve gastrectomy, potentially reflecting altered reward circuits or symptom substitution [31].

### 4.6 Expectations and satisfaction

Approximately half of participants reported congruence between preoperative expectations and postoperative outcomes; the remainder experienced outcomes exceeding or falling short across domains including weight loss, complications, and health status.

In literature, patients often view surgery as a definitive solution, expecting unrealistic superior outcomes regarding weight loss magnitude. Beliefs as surgery will "solve my problems," "enhance appearance," or "guarantee success" are common and negatively influence postoperative outcomes, contributing to frustration and psychological decompensation [32]. This study confirms that expectation-outcome discrepancies correlate with reduced satisfaction and decreased willingness to undergo surgery again. Addressing unrealistic expectations in the preoperative phase is recommended [32].

Almost 80% would reconsider surgery, with satisfaction independent of weight loss or reoperation rate but significantly associated with weight stability and expectation-outcome congruence. This pattern aligns with long-term literature showing no association between satisfaction and absolute weight loss, underscoring the multifaceted nature of postoperative satisfaction [15,20]. Satisfaction predictors evolve over time: weight loss and perceived health dominate at six months to two years, body dissatisfaction at one year [33], while our 19-year data identify weight stability as a critical long-term determinant.

Several factors may explain high satisfaction despite variable adherence and suboptimal outcomes. First, satisfaction may reflect relative improvement from preoperative state rather than achievement of ideal outcomes—even patients with substantial weight regain may experience meaningful gains compared to their pre-surgical condition. Second, patients may reframe expectations over time, shifting from weight-loss goals toward broader quality-of-life improvements. Third, having exhausted conservative treatments, patients may view surgery as their best available option regardless of imperfect outcomes. Finally, non-weight benefits including sustained comorbidity improvements, enhanced physical functioning, or psychosocial gains may persist independent of weight outcomes. The weight stability finding suggests that ongoing fluctuations diminish satisfaction. This warrants replication.

### 4.7 Encountered barriers and peer advice

Reported barriers encompassed eating and drinking, physical, psychological, and social domains. Some participants felt isolated and lonely due to inadequate understanding and support within their social networks, both regarding the initial decision to undergo surgery and during the long-term postoperative adaptation. This finding aligns with research indicating that social support is a critical factor in bariatric surgery outcomes, and highlights the importance of support systems into comprehensive bariatric care programs [34]. To our knowledge, no previous study examined patient-reported barriers, though reported challenges overlapped with studies investigating unmet expectations [32]. Barriers underrepresented in literature included dental issues [35] and endocrinological complications such as pancreatitis and gallstones [36], warranting broader examination of long-term consequences.

Peer advice ranged from recommendation to cautionary guidance and emphasized gathering comprehensive information from multiple sources, characterizing surgery as a weight loss tool rather than comprehensive solution. Recommendations highlighted awareness of emotional eating's impact, dietary guideline adherence, sustained effort for maintaining results, and social support value.

These patient recommendations align with our quantitative findings, highlighting three priority areas for long-term follow-up: sustained nutritional monitoring (given one-third minimal vitamin use), ongoing behavioral support for eating patterns (particularly snacking control), and weight stability monitoring with early intervention to prevent regain and dissatisfaction. However, our study was conducted before the advent of GLP-1 agonist therapy for post-bariatric weight management. Given the emerging role of these medications in managing weight regain after bariatric surgery, future studies should examine their use, effectiveness, and patient perspectives in long-term follow-up cohorts.

Our findings align with our previously published 5-year study [11], confirming persistent eating behavior challenges, vitamin non-adherence and satisfaction independent of absolute weight loss. Novel insights from the 19-year follow-up include: (1) weight stability—rather than absolute loss—as the key satisfaction predictor, (2) expectation-outcome congruence as critical for satisfaction, and (3) patient wisdom characterizing surgery as a 'tool requiring lifelong effort' rather than cure.

### 4.8 Limitations

This study contributes to the limited body of long-term bariatric outcomes research, particularly from patient perspectives. Nevertheless, several limitations warrant acknowledgment.

First, contemporary procedures (gastric sleeve, gastric bypass) differ from those undergone by participants. However, no evidence suggests modern procedures specifically impede or facilitate postoperative lifestyle adaptation. Additionally, LAGB management remains relevant in many bariatric practices [37]. Postoperative guidelines remain largely unchanged, and long-term behavioral studies remain scarce. Therefore, these findings likely provide meaningful insights into long-term postoperative behaviors.

Second, response rate was relatively low, a common challenge in long-term follow-up studies. These exploratory findings require replication in future research.

Finally, self-report bias (overreporting positive outcomes, underreporting negative outcomes) cannot be excluded. However, current findings align with other long-term studies incorporating objective weight assessment. Moreover, this study prioritized patient perspectives to capture subjective experiences regarding benefits and risks, which makes self-report essential.

## 5 Conclusions

Long-term adherence to healthy lifestyle behaviors and dietary guidelines was inconsistent. Most patients would reconsider bariatric surgery. Satisfaction did not depend on weight loss or reoperation, while weight instability and expectation-outcome discrepancies reduced surgical reconsideration likelihood. Defining success solely through weight loss outcomes and comorbidity reduction may inadequately represent patient perspectives. We advocate for lifelong follow-up to optimize postoperative results.

## Disclosures

The authors declare that they have no known competing financial interests or personal relationships that could have appeared to influence the work reported in this paper.

## Clinical takeaways

• After 19 years, satisfaction was high irrespective absolute weight loss. Weight stability, not absolute weight loss, is what matters most for long-term patient satisfaction
• Discrepancies between preoperative expectations and actual outcomes were key predictors of dissatisfaction
• One-third of patients discontinue vitamin supplements long-term, risking preventable nutritional complications

## Author contribution

Conceptualization, methodology, formal analysis and data curation, including writing the original draft was by G. Konings. M. Drukker was responsible for the formal analysis. M. Drukker, R. Severeijns and Rudolf Ponds were also responsible for review and editing. All authors approved the final submission and publication.

## Ethics

This submission represents original work and has not been published, or for consideration for publication, elsewhere. This study was carried out in accordance with The Code of Ethics of the World Medical Association (Declaration of Helsinki) for experiments involving humans.

## Ethical statement Obesity Research and Clinical Practice

All authors of the article “Long Term Patient Perspectives Following all types of Bariatric Surgery: A 19-Year Follow-Up Study” declare that:

1. There are no conflicts of interest and written informed consent was
  obtained from all individual participants included in the study.
2. We have read and have abided by the statement of ethical standards for manuscripts submitted to the Obesity Research & Clinical Practice. Therefor, all human subjects were conducted in accordance with the Declaration of Helsinki and all procedures were carried out with the adequate understanding and written consent of the subjects.
3. Formal approval has been obtained from the human subjects review board of the Academic Hospital of Maastricht and can be provided upon request.

## Authors agreement

Regarding the manuscript “Long Term Patient Perspectives Following All Types of Bariatric Surgery: A 19-Year Follow-Up Study”, the authors G. Konings, M. Drukker, R. Severeijns and R. Ponds certify and declare the following:

• All authors have reviewed and approved the final version of this manuscript and agree to its submission for publication.
• Each author confirms that the manuscript represents honest and original work. The article is not under consideration for publication elsewhere in whole or in part.
• The corresponding author has been authorized by all co-authors to act on their behalf in all matters related to the publication of this manuscript.

## Declaration of artificial intelligence

The authors declare that no part of this manuscript was written or edited using any AI or AI products including figures or tables. AI-based grammar checking tools were used solely for language quality assurance. After using this tool, the authors reviewed and edited the content as needed and take full responsibility for the content of the publication.

## Source of funding

The authors declare that they have no conflict of interest. This research did not receive any specific grant from funding agencies in the public, commercial, or not-for-profit sectors.

## References

[1] Kim J.C., Kim M.G., Park J.K., Lee S., Kim J., Cho Y.S. (2023). Outcomes and adverse events after bariatric surgery: an updated systematic review and meta-analysis, 2013-2023. J Metab Bariatr Surg.

[2] O'Brien P.E., Hindle A., Brennan L., Skinner S., Burton P., Smith A. (2019). Long-term outcomes after bariatric surgery: a systematic review and meta-analysis of weight loss at 10 or more years for all bariatric procedures and a single-centre review of 20-Year outcomes after adjustable gastric banding. Obes Surg.

[3] Arterburn D.E., Telem D.A., Kushner R.F., Courcoulas A.P. (2020). Benefits and risks of bariatric surgery in adults: a review. JAMA.

[4] Courcoulas A.P., Daigle C.R., Arterburn D.E. (2023). Long term outcomes of metabolic/bariatric surgery in adults. Br Med J.

[5] Konttinen H., Peltonen M., Sjöström L., Carlsson L., Karlsson J. (2015). Psychological aspects of eating behavior as predictors of 10-y weight changes after surgical and conventional treatment of severe obesity: results from the Swedish obese subjects intervention study. Am J Clin Nutr.

[6] Athanasiadis D.I., Martin A., Kapsampelis P., Monfared S., Stefanidis D. (2021). Factors associated with weight regain post-bariatric surgery: a systematic review. Surg Endosc.

[7] Sheets C.S., Peat C.M., Berg K.C., White E.K., Bocchieri-Ricciardi L., Chen E.Y. (2015). Post-operative psychosocial predictors of outcome in bariatric surgery. Obes Surg.

[8] Sherf Dagan S., Goldenshluger A., Globus I., Schweiger C., Kessler Y., Kowen Sandbank G. (2017). Nutritional recommendations for adult bariatric surgery patients: clinical practice. Adv Nutr.

[9] Coulman K.D., Howes N., Hopkins J., Whale K., Chalmers K., Brookes S. (2016). A comparison of health Professionals' and Patients' views of the importance of outcomes of bariatric surgery. Obes Surg.

[10] Konings G., Drukker M., Severeijns R., Ponds R. (2023). The complexity of obesity-related health problems after bariatric surgery: the patient perspective. Obesity pillars.

[11] Konings G., Drukker M., Severeijns R., Ponds R. (2023). The complexity of obesity-related health problems after bariatric surgery: the patient perspective. Obesity Pillars.

[12] Mendes F.H., Viterbo F. (2017). Defining “Weight Stability” for post-bariatric body contouring procedures. Aesthetic Plast Surg.

[13] Sjöström L., Narbro K., Sjöström C.D., Karason K., Larsson B., Wedel H. (2007). Effects of bariatric surgery on mortality in Swedish obese subjects. N Engl J Med.

[14] Bryant E.J., Malik M.S., Whitford-Bartle T., Waters G.M. (2020). The effects of bariatric surgery on psychological aspects of eating behaviour and food intake in humans. Appetite.

[15] Tolvanen L., Christenson A., Surkan P.J., Lagerros Y.T. (2022). Patients' experiences of weight regain after bariatric surgery. Obes Surg.

[16] van Wezenbeek M.R., Smulders J.F., de Zoete J.P.J.G.M., Luyer M.D., van Montfort G., Nienhuijs S.W. (2015). Long-term results of primary vertical banded gastroplasty. Obes Surg.

[17] Straatman J., Demirkiran A., Harlaar N.J., Cense H.A., Jonker F.H.W. (2023). The impact of reoperations following bariatric surgery on mid-term outcomes. Obes Surg.

[18] de Lourdes M., Cerqueira L., Pinto-Bastos A., Marôco J., Palmeira L., Brandão I. (2021). Understanding uncontrolled eating after bariatric surgery: the role of excessive skin and body image shame. J Clin Med.

[19] Lynch A. (2016). “When the honeymoon is over, the real work begins:” Gastric bypass patients' weight loss trajectories and dietary change experiences. Soc Sci Med.

[20] Nogué M., Nogué E., Molinari N., Macioce V., Avignon A., Sultan A. (2019). Intuitive eating is associated with weight loss after bariatric surgery in women. Am J Clin Nutr.

[21] Engin A. (2024). Bariatric surgery in obesity: metabolic quality analysis and comparison of surgical options. Adv Exp Med Biol.

[22] de Zwaan M., Marschollek M., Allison K.C. (2015). The night eating syndrome (NES) in bariatric surgery patients. Eur Eat Disord Rev.

[23] Gasmi A., Bjørklund G., Mujawdiya P.K., Semenova Y., Peana M., Dosa A. (2022). Micronutrients deficiences in patients after bariatric surgery. Eur J Nutr.

[24] Kim T.Y., Kim S., Schafer A.L., Feingold K.R., Ahmed S.F., Anawalt B., Blackman M.R., Boyce A., Chrousos G. (2000). Endotext. South Dartmouth (MA): MDText.com, inc. Copyright © 2000-2025.

[25] Al-Najim W., Docherty N.G., le Roux C.W. (2018). Food intake and eating behavior after bariatric surgery. Physiol Rev.

[26] Qadhi A.H., Almuqati A.H., Alamro N.S., Azhri A.S., Azzeh F.S., Azhar W.F. (2023). The effect of bariatric surgery on dietary behaviour, dietary recommendation adherence, and micronutrient deficiencies one year after surgery. Prev Med Rep.

[27] Chen L., Chen Y., Yu X., Liang S., Guan Y., Yang J. (2024). Long-term prevalence of vitamin deficiencies after bariatric surgery: a meta-analysis. Langenbecks Arch Surg.

[28] Johansson K., Svensson P.A., Söderling J., Peltonen M., Neovius M., Carlsson L.M.S. (2021). Long-term risk of anaemia after bariatric surgery: results from the Swedish obese subjects study. Lancet Diabetes Endocrinol.

[29] Kobylińska M., Antosik K., Decyk A., Kurowska K. (2022). Malnutrition in obesity: is it possible?. Obes Facts.

[30] Capelo Vides M., Campello de Oliveira M., Lassi D.L.S., Malbergier A., Florio L., de Azevedo-Marques Périco C. (2023). Bariatric surgery and its influence on alcohol consumption: differences before and after surgery - a systematic review and meta-analysis. Int Rev Psychiatr.

[31] Nance K., Acevedo M.B., Pepino M.Y. (2020). Changes in taste function and ingestive behavior following bariatric surgery. Appetite.

[32] Hering I., Stier C., Seyfried F. (2018). [Bariatric surgery: expectations and therapeutic goals-a contradiction?]. Chirurgie.

[33] Gaudrat B., Florent V., Andrieux S., Rousseau A. (2021). "I Want to Lose Weight and it Has to Be Fair": predictors of satisfaction after bariatric surgery. Obes Surg.

[34] Jawara D., Alagoz E., Lauer K.V., Voils C.I., Funk L.M. (2024). Exploring social support dynamics after bariatric surgery: insights from patients and providers. J Surg Res.

[35] Ferraz A.X., Gonçalves F.M., Ferreira-Neto P.D., Santos R.S., Guariza-Filho O., Zeigelboim B.S. (2023). Impact of bariatric surgery on oral health: a systematic review and meta-analysis. Clin Oral Invest.

[36] Bischoff S.C., Ockenga J., Eshraghian A., Barazzoni R., Busetto L., Campmans-Kuijpers M. (2023). Practical guideline on obesity care in patients with gastrointestinal and liver diseases - joint ESPEN/UEG guideline. Clin Nutr.

[37] Benson-Davies S., Rogers A.M., Huberman W., Sann N., Gourash W.F., Flanders K. (2022). American society of metabolic and bariatric surgery consensus statement on laparoscopic adjustable gastric band management. Surg Obes Relat Dis.

